# Endoscopic Treatment Using the Padlock Clip System for Rectourethral Fistula After Prostatectomy

**DOI:** 10.7759/cureus.33250

**Published:** 2023-01-02

**Authors:** Daniel De Barcellos Azambuja, Bruna Oliveira Trindade, Sarah Bueno Motter, Gabriela Rangel Brandão, Radames lacava Schramm, Giovani Thomaz Pioner

**Affiliations:** 1 Colorectal Surgery, Santa Casa de Misericórdia de Porto Alegre, Porto Alegre, BRA; 2 Medical School, Federal University of Health Sciences of Porto Alegre, Porto Alegre, BRA; 3 Urology, Santa Casa de Misericórdia de Porto Alegre, Porto Alegre, BRA

**Keywords:** fistula management, endoscopic approach, padlock clip, prostatectomy, rectourethral fistula

## Abstract

We present a case of a 68-year-old man with a rectourethral fistula (RUF) successfully treated with a unique endoscopic approach using the Padlock Clip system (Steris, Basingstoke, UK). This is a complex case of a patient who, after radical prostatectomy, continued to show several complications, including fistulas and relapses. Our work aims to enhance the literature with our technique and to help the scientific community in future RUF cases. Our case stands out because this therapeutic approach has not yet been described in the literature as a possible endoscopic treatment of RUF. Therefore, our topic description is essential to assist future similar cases.

## Introduction

Rectourethral fistula (RUF) is a rare postoperative complication for benign prostatic enlargement or prostate cancer [1], with a limited incidence of 0.53% after prostatectomy [2]. Formation of a RUF is associated with the final step of prostatectomy surgery, i.e., ureterovesical anastomosis [2]. The patient frequently experiences rectal urinary leakage, fecaluria, and pneumaturia [1]. Rectal examination typically identifies the fistula site, and cystourethroscopy, cystourethrography, or a contrast rectus investigation reveals the fistula [1].

Surgery should be considered for all oncological patients deemed fit for it who understand the surgical risks due to the significant morbidity and mortality associated with complications, such as fistula [3]. RUF surgical management is always difficult [2]. There currently needs to be an agreement on the best technique for repairing [4]. We can now explore the possibility of endoscopic closure for intestinal fistulas because of recent advancements in endoscopic procedures [5]. Over 40 approaches have been described in the literature, ranging from transanal endoscopic microsurgery to transabdominal surgery [4].

The current study aims to present the RUF case of a 68-year-old man successfully treated endoscopically using the Padlock Clip system (Steris, Basingstoke, UK).

## Case presentation

In February 2020, a 68-year-old man underwent a laparoscopic radical prostatovesiculectomy, pelvic lymphadenectomy, and rectum raffia due to prostate cancer, stage IIA cT2a. He also had systemic arterial hypertension and unipolar depression as comorbidities. The patient was on simvastatin, losartan, amlodipine, azathioprine, and bupropion for his comorbidities at the time of surgery.

In March 2020, the patient noticed urine output from the anorectal region. For that reason, he searched for medical appointments. The doctor suggested the insertion of an indwelling urinary catheter (IUC) and the evaluation of the fistula by a coloproctologist. Two months after that, the volume of urine output increased, and he observed a urine overflow on the IUC drainage tube. During this episode, he went to the emergency service, and the medical team suspected obstruction of the IUC. The patient denied fever, abdominal pain, and other inflammatory symptoms at that time. The IUC was exchanged, and the patient was instructed to continue the follow-up with the coloproctologist.

**Figure 1:**
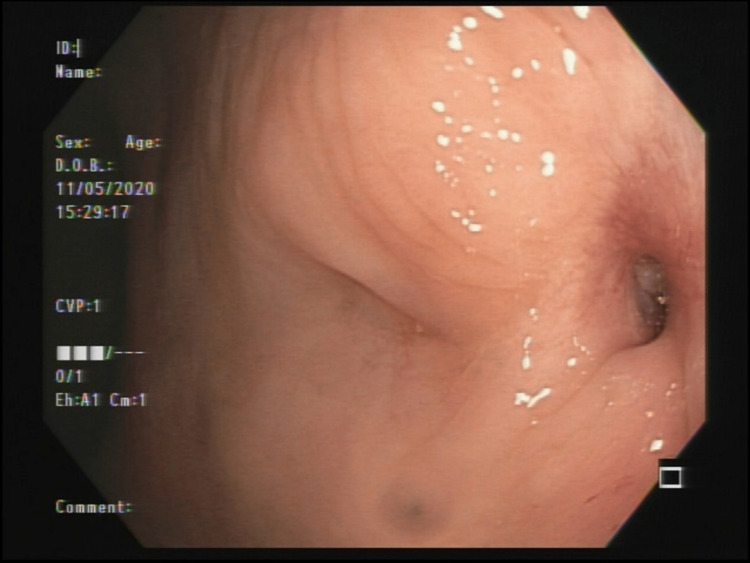
Endoscopic rectourethral fistula orifice discovery localized on the rectum and anterior anal canal

In May 2020, our coloproctology team examined the patient. We detected that the anorectal area leaked urine when IUC was removed or obstructed. Due to the acute character of symptoms immediately after the surgical procedure, we discarded the differential diagnosis of congenital anorectal malformations or secondary to inflammatory bowel disease. Thus, we considered urorectal fistula following the treatment of prostate cancer as the primary diagnostic hypothesis. A colonoscopy was performed to confirm the diagnosis. We found a fistulous orifice in the transition of the rectum and anterior anal canal with urethral communication (Figure 1). At the end of the same month, a sigmoidostomy was placed to decrease intraluminal pressure. On July 1, 2020, the patient was discharged with an excellent surgical response from the sigmoidostomy.

On August 10, 2020, the patient returned for reevaluation with a urethrocystography showing a fistula recurrence. The team decided to wait 30 days to perform other interventions to observe if, with more time, the fistula would regress. On September 17, 2020, a new fistula repair was performed by a sagittal posterior approach (Kraske procedure). After one month of hospitalization, the patient was discharged with a urethrocystography showing healed fistula, and the team advised repeating the exam as a follow-up.

A control exam on January 18, 2021, showed fistula return and occasional urine output. Also, the patient reported doing hyperbaric oxygen therapy sessions with symptom relief. Due to the fistula’s recurrence, we recommended endoscopic clipping and the application of fibrin glue as the surgical option, but the patient chose not to do it. On May 5, 2021, the patient returned to our hospital and agreed to do the procedure. We observed him after 15 days and then every 30 days, followed up after that surgery. On August 19, 2021, the patient had no signs of fistula relapse, and we reversed the colostomy. During a follow-up visit on October 10, 2021, new urethrocystography showed signs of fistula persistence. We advise that a new procedure could be performed in January 2022. The patient, however, returned only on May 23, 2022, with urine output from the anorectal region. The patient was unwilling to undergo any new procedures due to complications and was against the colostomy possibility.

**Figure 2:**
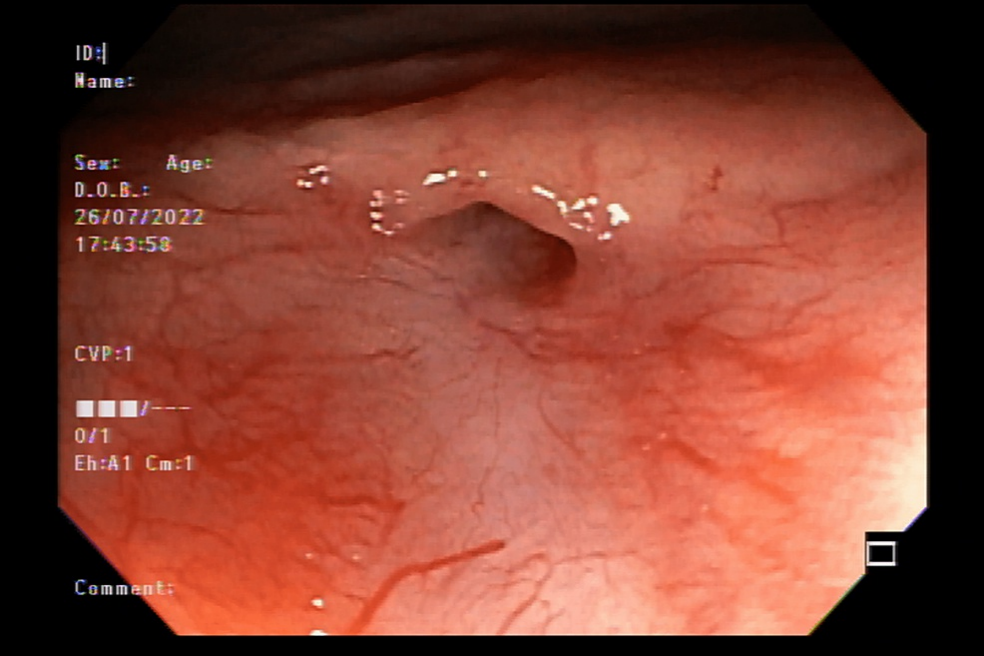
Colonoscopy to evaluate the rectourethral fistula orifice recurrence

**Figure 3:**
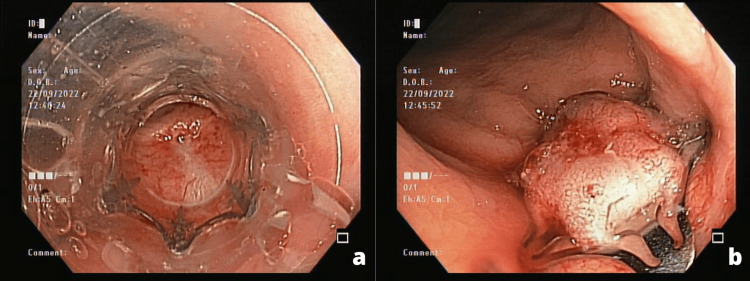
Endoscopic approach of fistula closure using a Padlock Clip (a) Endoscopic identification of the fistula location. (b) Endoscopic suctioning of the fistula and a Padlock Clip applied at the fistula site.

On a return appointment on July 14, 2022, we requested a new colonoscopy (Figure 2) to assess the possibility of further surgical intervention. After following recommendations for fistula repair, the colorectal and urology team performed a multidisciplinary round and a literature review to develop a new solution. Due to the continued recurrence, we chose to innovate with an endoscopic clip commonly used for gastrointestinal fistula called Padlock Clip. We did this procedure on September 23, 2022 (Figure 3), and the patient recovered with no complications in the postoperative period. Follow-up visits continue every month to evaluate reoccurring and symptomatology, both negative until December 2022.

## Discussion

RUF as a complication of radical prostatectomy is rare [1]. In addition to significant physiologic, biochemical, and infectious changes, the fistula can result in psychological suffering [6]. Thus, a quick treatment plan is essential for a good outcome [6]. Treatment includes several possibilities [7], including conservative therapies that monitor spontaneous fistula closure. Due to their anatomical placement and the involvement of the urinary and anal sphincters, RUFs are challenging [2]. Little is known about the best treatment for post-prostatectomy fistulas due to the lack of randomized studies and the absence of standardized action protocols [2]. Therefore, we present a rare complication treated endoscopically with the Padlock Clip.

The case that we report above is complex. Our patient, after radical prostatectomy, presented several complications, including fistulas and relapses. With the failure of other techniques and therapeutic approaches, the novel Padlock was proposed. The use of over-the-scope clips for gastrointestinal tract fistulas has already been proven viable, safe, and effective [8,9]. The new Padlock Clip is an innovative and versatile alternative. It has been used and successfully described in the literature by some reports or case series for use in gastrointestinal fistula [10], such as to stop recurrent bleeding and to close perforations or fistula at different levels [6]. For our patient, the Padlock Clip therapeutic result has been successful so far. There was no failure of the method or relapse in the short term (three months). We highlighted the follow-up continues to confirm a long-term success, as this patient has multiple recurrences.

Our case stands out because this therapeutic has not yet been described in the literature as a possible endoscopic treatment of RUF. We found two similar cases in the previous literature. However, they were both female cases with fistula communicating with the bladder [6]. Velayos et al. [6] treated a fistula in the sigmoid colon, but it was non-visible through colonoscopy and a consequence of an inflammatory-associated disease. Chiam et al. [5] also used the same endoscopic device to close the radiation-induced colovesical fistula. Those examples confirm the uniqueness and relevance of the present case.

We described this therapeutic approach to improve the literature using our method and to assist the scientific community in cases similar to this one in the future. However, due to the scarcity of evidence in the literature regarding this specific technique, especially in the long term, we reinforce that surgeons should still decide to use this procedure on a case-by-case basis. Therefore, with the scientific production of new reports and more extensive studies, it will be possible to create future evidence for routine use in these cases.

## Conclusions

Although RUF is a rare complication of radical prostatectomy, it deserves further studies, as it brings pathological complications to the patient and directly affects their quality of life. Our unique case shows a new therapeutic possibility managed with a multidisciplinary team; therefore, the description of the topic is necessary to enhance the literature with our technique and help the scientific community in future similar cases.
